# Varicella zoster virus-induced neurological disease after COVID-19 vaccination: a retrospective monocentric study

**DOI:** 10.1007/s00415-021-10849-3

**Published:** 2021-11-01

**Authors:** Samir Abu-Rumeileh, Benjamin Mayer, Veronika Still, Hayrettin Tumani, Markus Otto, Makbule Senel

**Affiliations:** 1 Department of Neurology, University of Ulm, Ulm, Germany; 2 Department of Neurology, Martin-Luther-University Halle-Wittenberg, Halle (Saale), Germany; 3 Institute of Epidemiology and Medical Biometry, Ulm University, Ulm, Germany; 4 Fachklinik für Neurologie Dietenbronn, Schwendi, Germany

**Keywords:** COVID-19, SARS-CoV-2, Varicella, Zoster, Vaccination, Vaccine

## Abstract

The description of every possible adverse effect or event related to vaccines is mandatory during the ongoing worldwide COVID-19 vaccination program. Although cases of cutaneous varicella zoster virus (VZV) reactivation after COVID-19 vaccination have been increasingly reported in literature and database sets, a description of VZV-induced neurological disease (VZV-ND) is still lacking. In the present study, we retrospectively evaluated patients admitted to our clinic and diagnosed with VZV-ND during the COVID-19 vaccination campaign (January–April 2021) and in the same months in the previous two years. We identified three patients with VZV-ND after COVID-19 vaccination and 19 unvaccinated VZV-ND cases as controls. In the case–control analysis, the two groups showed no difference in clinical features, results of diagnostic investigations, and outcome. Thus, VZV reactivation with neurological involvement might be a possible event triggered by COVID-19 vaccination, but the benefit following COVID-19 vaccination overcomes significantly the potential risk associated with a VZV reactivation.

## Introduction

To date, EMA approved in EU four vaccines against coronavirus disease 19 (COVID-19), namely Oxford/AstraZeneca chimpanzee adenovirus-vectored COVID-19 vaccine (ChAdOx1), Pfizer/BioNTech BNT162b2, Moderna mRNA-1273, and Johnson & Johnson/Janssen Ad26.COV2.S [1]. Despite the several benefits of vaccination in terms of public health, vigilance and safety monitoring of its side effects are mandatory. According to the Centers for Disease Control and Prevention (CDC) Vaccine Adverse Event Reporting System (VAERS) database, the most common adverse effects after COVID-19 vaccines are transient, including injection site pain, fever, and headache [2, 3]. Furthermore, cases of cutaneous varicella zoster virus (VZV) reactivation after COVID-19 vaccination have been reported in literature and database sets [2, 5], but a definitive causal relationship is still to be confirmed.

VZV or Herpes zoster virus is a human neurotropic herpes virus. After primary infection (varicella), the virus becomes latent in neurons of cranial nerve ganglia or dorsal root ganglia [6–8]. Due to a decline in VZV cell-mediated immunity (e.g. age-related immunosenescence or immunosuppression) the virus may reactivate causing zoster and/or several neurological manifestations which may also develop without rash (sine herpete), such as cranial nerve palsies (e.g. Ramsay-Hunt syndrome), meningitis, encephalitis, (poly)radiculitis, cerebellitis, myelopathy, vasculopathy and postherpetic neuralgia [6–8]. Thus, the burden of VZV disease is relevant, with more than 90% of the world population harboring a latent virus and more than 50% with a reactivation by 85 years of age [8]. The diagnosis of VZV-induced neurological disease (VZV-ND) relies on the detection of VZV-DNA by PCR and/or intrathecal synthesis of anti-VZV IgG and IgM in the cerebrospinal fluid (CSF)[7]. The gold standard treatment is acyclovir. Among triggers for VZV reactivation, vaccines are rarely reported, but a vaccine-induced immunomodulatory mechanism might be potentially involved [9, 10]. To date, there are cases of VZV reactivation concomitant with severe acute respiratory syndrome coronavirus type 2 (SARS-CoV-2) infection [11] but no description of VZV-ND after COVID-19 vaccination.

Taking into account all these issues, in the present study, we assessed the clinical features and results of diagnostic investigations of VZV-ND in a cohort of subjects during the COVID-19 vaccination campaign to evaluate a possible association between COVID-19 vaccination and VZV reactivation with neurological involvement.

## Methods

### Subjects

We performed a retrospective study at the Neurology Department of Ulm University Hospital (Germany). We identified all patients who were admitted to our clinic with a diagnosis of VZV-ND in the periods January–April 2021 (during COVID-19 vaccination), and we included a control groupwith the same diagnosis and recruited in the periods January–April 2019 and January–April 2020 (before COVID-19 vaccination). For the case–control analysis, we compared cases who received a vaccination against COVID-19 within 6 weeks before clinical onset [12] and unvaccinated subjects (diagnosed in 2019, 2020 and 2021). The local ethics committee of the University of Ulm approved this study (ethics approval number 236/21).

### Diagnosis

All cases with VZV-ND fulfilled the following criteria: (1) clinical picture of meningitis, encephalitis, meningoencephalitis, (poly) radiculitis, cerebellitis, myelitis, central nervous system (CNS) vasculopathy, (poly)neuritis cranialis and/or Ramsay-Hunt syndrome with or without zoster (sine herpete) [6–8], (2) complete CSF analysis including cell count and cell differentiation, CSF/serum albumin ratio, oligoclonal IgG bands (OCB), lactate, intrathecal IgG, IgA, and IgM synthesis, and confirmed VZV etiology by CSF PCR and/or increased pathogen-specific CSF/serum antibody indices (AIs) [6–8]; (3) available brain magnetic resonance imaging (MRI) at the day of lumbar puncture (LP) and (4) hospitalization with documented clinical history.

### Statistical analysis

Statistical analysis was performed using IBM SPSS Statistics version 21 (IBM, Armonk, NY, USA). Due to the small sample size, descriptive results were generally expressed as the median and interquartile range (IQR) in the case of continuous variables, as well as absolute and relative frequencies otherwise. Statistical comparisons between COVID-19 vaccinated and unvaccinated VZV-ND cases were done using the Mann–Whitney U test and Fisher’s exact test, whereas a *p* < 0.05 was considered statistically significant in a fully explorative manner.

## Results

In the periods January–April 2019, January–April 2020, and January–April 2021, we identified 6, 7, and 9 patients, respectively, with a diagnosis of VZV-ND who were admitted to our department. In the period January–April 2021, 3 cases received the COVID-19 vaccine before clinical onset, whereas 6 subjects were unvaccinated.

### Features of unvaccinated VZV-induced neurological disease cases (Table 1)

**Table 1:** Features of unvaccinated VZV-induced neurological disease cases

*N*	19
Age median (IQR); min–max	53 (39–71); 28–86
Female *N* (%)	7 (36.84)
Diagnosis *N* (%)	
Meningitis sine herpete (total)	8 (42.11)
With CNS vasculopathy	1 (5.26)
With (poly)neuritis cranialis	2 (10.53)
Meningitis with zoster	4 (21.05)
Meningoradiculitis sine herpete	1 (5.26)
Encephalitis sine herpete	1 (5.26)
Ramsay-Hunt syndrome with zoster	3 (21.05)
Ramsay-Hunt syndrome sine herpete	1 (5.26)
Trigeminal neuritis with zoster	1 (5.26)
Main clinical features *N* (%)	
Headache and/or facial pain	14 (73.68)
Facial palsy	4 (21.05)
Hearing and/or vestibular impairment	3 (15.79)
Other focal deficits	3 (15.79)
Radicular pain	2 (10.53)
Seizures	3 (15.79)
Fever	3 (15.79)
Nausea	3 (15.79)
Zoster *N* (%)	8 (42.11)
Previous COVID-19	1 case 1 year before
Immunodeficit N (%)	
Diabetes; IgA-deficit	2 (15.79); 1 (10.53)
Brain MRI	
Normal; inflammatory changes or other alterations	16 (84.21); 3 (15.79)
EEG	
Normal; abnormal	6/7 (85.71); 1/7 (14.29)
Time between onset and LP days Median (IQR)	6 (2–8)
Leukocyte count (/µL) (Norm < 5)	
Median (IQR); min–max	176 (60–453); 12- 635
Pleocytosis with 50–80% lymphocytes N (%)	9 (47.37)
Pleocytosis with > 80% lymphocytes N (%)	10 (52.63)
Protein (mg/L) (Normal range 200–500) Median (IQR)	819 (601–1845)
Lactate (mmol/L) (Normal range 1.3–2.7) Median (IQR)	2.41 (1.95–3.20)
Increased *N* (%)	8 (42.11)
CSF/serum albumin ratio × 10–3 Median (IQR)	13.2 (9.7–29.8)
Blood-CSF-barrier dysfunction *N* (%)	14 (73.68)
Positive oligoclonal IgG bands	
In first LP N (%); in follow-up LP *N* (%)	2 (10.53); 7/13 (53.85)
Intrathecal IgG, IgA, IgM synthesis	
In the first LP N (%); In the follow-up LP *N* (%)	0 (0); 0 (0)
VZV-DNA-PCR in the first LP^a^	
Positive; borderline *N* (%)	10/17 (58.82); 2/17 (11.76)
Negative *N* (%)	5/17 (29.41)
VZV-AI > 1.5 (Normal < 1.5)	
In the first LP N (%); In the follow-up LP N (%)	7/16 (43.75); 11/13 (84.62)
CXCL13 (pg/ml) (Normal < 10) Median (IQR)	50 (10–240)
Increased *N* (%)	10/12 (83.33)

*AI* antibody indices; *COVID*-*19* coronavirus disease 19; *CNS* central nervous system; *CSF* cerebrospinal fluid; *CXCL13* chemokine ligand 13; *EEG* electroencephalogram; *IQR* interquartile range; *LP* lumbar puncture; *MRI* magnetic resonance imaging; *PCR* polymerase chain reaction; *VZV* varicella zoster virus

^a^In second LP when not performed in the first

The 19 unvaccinated VZV-ND subjects showed a prevalence of males (63%) and a median age of 53 (IQR 39–71) years. The most common VZV-ND manifestations were meningitis (*n* = 12, 63%) with (*n* = 4, 21%) and without zoster (sine herpete) (*n* = 8, 42%) and Ramsay-Hunt syndrome (*n* = 4, 22%). In all subjects, the CSF analysis revealed a lymphocytic pleocytosis (min 12–max 635/µL) with increased protein levels. Increased lactate and CSF-blood-dysfunction were found in 42% and 74% cases, respectively. All cases showed no intrathecal Ig synthesis (in the Reiber diagrams for IgM, IgA, and IgG), whereas 11% and 54% subjects had positive oligoclonal IgG bands at first and follow-up LP, respectively. A positive or borderline VZV-DNA-PCR was detected in 71% patients. In the follow-up LP, 85% cases demonstrated an increased VZV-AI. CSF CXCL13 was elevated (> 10 pg/ml) in 10 out of 12 tested patients. All cases were treated with acyclovir and showed a complete recovery or a clinical improvement.

### Features of vaccinated VZV-ND cases and comparison with unvaccinated subjects (Tables 2 and 3)

**Table 2:** Demographic, clinical characteristics and results of diagnostic investigations of vaccinated VZV-ND cases

	Case 1	Case 2	Case 3
Age	82	70	63
Sex	Female	Female	Male
Diagnosis	VZV meningitis with Ramsay-Hunt syndrome sine herpete	VZV meningitis sine herpete	VZV meningoradiculitis sine herpete
Clinical symptoms at admission	Left peripheral facial palsy, headache, facial pain, nausea and vertigo	Headache, left facial pain and nausea	Proximal pain in lower back and lower limbs, headache
Neurological examination	Left peripheral facial palsy, left vestibular defect	Unremarkable	Dysreflexia in the lower extremities
Other clinical features	No typical skin, ear and eye lesions	No typical skin lesions	No typical skin lesions
COVID-19 vaccine type	BNT162b2	ChAdOx1	ChAdOx1
Administration	1st dose	1st dose	1st dose
Time between vaccination and clinical onset (days)	12	31	41
Serum SARS-CoV-2 IgA	NA	Negative	Negative
Serum SARS-CoV-2 IgG	NA	Positive	Positive
Previous varicella	In childhood	Not remembered	In childhood
Previous zoster	No	No	No
Previous zoster vaccination	No	No	No
Previous COVID-19	No	No	No
Comorbidities	Coronaropathy, hypertension, dyslipidemia, obesity	None	None
Brain MRI	Normal	Normal (no signs of trigeminal nerve involvement)	Normal
Brain CT	No signs of sinusitis and otitis	NA	NA
Spinal MRI	NA	NA	Normal (no signs of radiculopathy, myelopathy and lesions of conus/ cauda equina)
EEG	NA	Normal	Normal
Routine blood investigations	Normal	Normal	Normal

*COVID-19* coronavirus disease 19; *CT* computer tomography; *EEG* electroencephalogram; *MRI* magnetic resonance imaging; *NA* not available; *SARS-CoV-2* severe acute respiratory syndrome coronavirus 2; *VZV* varicella zoster virus

**Table 3:** CSF results of vaccinated VZV-ND cases

LP	Case 1		Case 2		Case 3		
	first	second	first	second	first	second	third
Days from clinical onset	7	17	5	9	5	11	17
Leukocyte count (/µL) (< 5)	325	46	34	63	1124	341	111
Lymphocytes (%)	70	75	88	85	NA	70	67
Activated lymphocytes (%)	15	9	8	7	NA	19	9
Plasmacells (%)	6	0	2	1	NA	5	3
Monocytes (%)	9	14	2	7	NA	6	11
Protein (mg/L) (Normal range 200–500)	1256	985	415	382	1635	546	509
Lactate (mmol/L) (Normal range 1.3–2.7)	3.79	3.27	1.5	1.46	2.62	2.15	1.91
CSF/serum albumin ratio × 10^–3^	17.7	14.6	5.3	5.2	22.7	9.5	7.2
Blood-CSF-barrier dysfunction	Yes	Yes	No	No	Yes	Yes	NA
oligoclonal IgG Bands	Negative	Positive	Negative	Negative	Negative	Positive	NA
Intrathecal IgG, IgA, IgM synthesis	No	IgA 20.2%	No	No	No	No	No
VZV-DNA-PCR	NA	Negative	NA	Negative	Positive	Borderline	Negative
VZV-AI (Normal < 1.5)	8.0	4.2	0.9	5.2	1.3	2.6	NA
CXCL13 (pg/ml) (Normal < 10)	366	45	NA	18	63	45	NA
CSF SARS-CoV2-PCR	NA	Negative	NA	Negative	Negative	NA	NA
CSF SARS-CoV-IgA and IgG	NA	NA	NA	Negative	Negative	NA	NA
Investigations with normal results	HSV 1,2, bacteria, fungi, Borrelia b., TBE		HSV 1,2, CMV, EBV, HHV6, bacteria, fungi, Borrelia b., TBE, Mumps, Rubella, HIV, HAV, HBV, HCV, tubercolosis, CNS-autoantibodies, autoimmune screening		HSV 1,2, CMV, EBV, HHV6, bacteria, fungi, Borrelia b., TBE		

*AI* antibody indice; *CMV* cytomegalovirus; *CSF* cerebrospinal fluid; *CXCL13* Chemokine ligand 13; *EBV* Epstein-Barr virus; *HAV, HBV, HCV* hepatitis A, B, C virus; *HHV6* human herpesvirus 6; *HIV* human immunodeficiency virus; *HSV* herpes simplex virus; *LP* lumbar puncture; *MRI* magnetic resonance imaging; *NA* not available; *PCR* polymerase chain reaction; *SARS-CoV-2* severe acute respiratory syndrome coronavirus 2; *TBE* Tick-borne encephalitis; *VZV* varicella zoster virus

All vaccinated VZV-ND cases showed neurological manifestations without zoster (sine herpete) (Table 2). A lymphocytic pleocytosis was disclosed in all patients. Protein levels were increased in 2/3 patients. Lactate was within the normal range in 2/3 patients. Oligoclonal IgG bands were positive in follow-up LPs in 2 out of 3 patients. VZV-PCR was positive in 1 out of 3 patients, whereas VZV-AI was increased in all patients. At variance with cases 2 and 3, in case 1 we did not detect a progressive increase of VZV-AI between two consecutive LPs. Nevertheless, given the typical features of Ramsay-Hunt syndrome (e.g., facial and vestibulocochlear nerve involvement), a diagnosis of possible VZV-ND was made. All subjects showed variably high levels of CSF CXCL13 (Table 3). During the hospitalisation and under therapy with acyclovir, clinical picture and CSF biochemical parameters improved significantly in all patients. In detail, in patients 2 and 3 the symptoms completely disappeared, while in patient 1 we observed an improvement of facial palsy and vestibular defect, which further improved after rehabilitation.

All fully explorative comparisons revealed no statistically significant difference between vaccinated and non-vaccinated patients regarding demographic (age, sex, time from onset to LP), CSF parameters (leukocyte count, protein, lactate, CSF/serum albumin ratio, increased VZV-AI, positive OCBs, positive/borderline VZV-DNA-PCR) and outcome. For MRI and EEG data, no comparison was possible due to the small sample size.

## Discussion

A vigilant reporting and a complete transparency in the description of every possible adverse effect related to vaccines represent an important chapter in public healthcare during an ongoing worldwide vaccination program.

In the present study, we retrospectively evaluated patients who were admitted to our clinic and had a diagnosis of VZV-ND during the COVID-19 vaccination campaign (January–April 2021) and in the same months in the previous two years. We found three patients who developed VZV-ND after COVID-19 vaccination. Given that age is the major risk factor for VZV reactivation in 90% of cases [13] and that none of our cases was immunosuppressed, one could argue that age might have played here a significant role. However, demographic features, including age, outcome, and results of a diagnostic investigation, did not differ between vaccinated and unvaccinated VZV-ND cases. From one side, this finding may suggest that the vaccine could have triggered or at least contributed to the virus reactivation. On the other side, the typical good outcome in most VZV-ND cases and the low case fatality [14] seem to characterise also VZV-ND after COVID-19 vaccination.

The BNT162b2 and ChAdOx1vaccine trials reported no cases of zoster or VZV-ND as adverse events among vaccine recipients [15–17]. Moreover, a recent study found no evidence for increased oropharyngeal reactivation of herpesviruses one week after BNT162b2 administration, arguing against a possible reactivation mechanism linked to the vaccination [18]. However, besides the short observation period (e.g., 1 week), the authors did not exclude that a symptomatic reactivation in trigeminal ganglion, facial nerve, or skin, might possibly occur with no increased oropharyngeal shedding [18]. Conversely, in both CDC VAERS and in the Yellow Card adverse reaction reporting scheme of Medicines and Healthcare products Regulatory Agency (MHRA) large databases sets, VZV-related complications after BNT162b2 and ChAdOx1 vaccines are increasingly observed [2, 4]. Accordingly, a very large epidemiological study showed that BNT162b2 vaccination was strongly associated with herpes zoster virus infection (e.g., VZV reactivation) with a risk ratio of 1.43 and a risk difference of 15.8 events per 100,000 persons at 6 weeks after administration [19]. Therefore, we would speculate that vaccines, including those against COVID-19, may induce an immunomodulatory effect with a temporary failure of VZV-specific T-cell response leading to VZV reactivation [10]. In this regard, several data support the role of T-cell-mediated immunity for the maintenance of latency of VZV [10, 20].

Given the monocentric nature and the small sample size of the study, we were not able to assess the possible association between COVID-19 vaccination and VZV-ND. Furthermore, based on the available data, it did not seem reasonable to calculate valid estimates for the incidence of VZV-ND in vaccinated and unvaccinated patients. In particular, an appropriate definition of the denominator is tough considering the current dynamic situation of COVID-19 vaccination rates. On another issue, one could argue that the first COVID-19 outbreak may have partially influenced the prevalence of VZV-ND observed in 2020, given the reported significant decrease in hospital admissions for neurological disorders (e.g., for stroke [21]). However, in 2019 (before the pandemic), a similar number of VZV-ND patients was admitted to our department. Nevertheless, we could not exclude that the temporal association between VZV reactivation and vaccination might represent only a pure coincidence. In this regard, we are aware that future epidemiological studies with longer follow-up periods may confirm or question the association between COVID-19 vaccination and VZV reactivation.

However, our data, together with concomitant observations, suggest that VZV reactivation might be a possible rare event triggered by COVID-19 vaccination and should aware clinicians to promptly consider this manifestation in the differential diagnosis and to rapidly start the specific antiviral treatment. Nevertheless, considering the morbidity and mortality associated with COVID-19, the benefit following COVID-19 vaccination overcomes significantly the potential risk associated with a VZV reactivation. However, in view of the hundreds of millions of individuals to be vaccinated against SARS-CoV-2, larger epidemiological studies are needed to definitely elucidate all these issues.

## Data Availability

Not applicable.
